# Morphometric Variations of Extraocular Muscles in a Caucasian Cohort: A Pilot Study on the Surgical Implications of Ethnic Diversity

**DOI:** 10.7759/cureus.104472

**Published:** 2026-03-01

**Authors:** Vinesh Mistry, Naila Ali, Karuna Katti

**Affiliations:** 1 Medicine, Anglia Ruskin University, Chelmsford, GBR; 2 Medicine, University of Nottingham, Nottingham, GBR; 3 Medicine, University of Lancaster, Lancaster, GBR

**Keywords:** capturing accurate race and ethnicity data, clinical and functional anatomy, extraocular muscles, eye surgery, ‏strabismus, strabismus surgery

## Abstract

Precise anatomical knowledge of the extraocular muscles (EOMs) is crucial, particularly for managing “slipped” or “lost” muscles in strabismus surgery. This pilot study provides the first morphometric data for Caucasian-specific, cadaveric orbits, revealing significant deviations from accepted anatomical norms. 6 Caucasian, formalin-embalmed orbits (86-92 years) were dissected using a superior orbitotomy approach at the Human Anatomy Unit, University of Birmingham. Recti and oblique lengths, insertion widths, limbal distances, and inter-recti distances were measured using a triple-observer, string-arc technique to account for scleral curvature. Data on muscle length, width, and limbal distance were compared with Thai, Mexican, Indian, and Taiwanese datasets using Welch’s t-tests; inter-recti spacing was assessed observationally. Significant deviations were observed across all assessed parameters, most notably in relation to the Spiral of Tillaux, with an ascending limbal distance order of lateral rectus (LR) < medial rectus (MR) < inferior rectus (IR) < superior rectus (SR). Relative to the compared East Asian cohorts, the Caucasian population exhibited significantly more posterior insertions in three of the four recti muscles (P < 0.05). Inter-recti distance comparison identified the IR-MR gap as consistently the narrowest surgical corridor. Morphometric data of the EOMs including the possible inter-ethnic differences suggested in this study, should be further established through additional research. Moreover, further studies are needed to validate the use of inter-recti distance as a useful surgical morphometric parameter that could guide more precise and tailored approaches in strabismus surgery.

## Introduction

Strabismus, commonly referred to as “crossed eyes,” is a prevalent visual disorder characterized by the misalignment of the eyes, causing them to point in different directions [1]. It typically develops during childhood and, if left untreated, can result in significant visual impairments such as amblyopia, diplopia, or loss of binocular vision [2]. Beyond visual consequences, strabismus can negatively impact an individual's self-esteem and quality of life due to its cosmetic and functional consequences. Surgical realignment of the extraocular muscles (EOMs) remains the primary treatment for strabismus. This procedure involves detaching and repositioning one or more muscles on the globe to correct ocular misalignment [2]. Despite advances in technique, surgical complications such as muscle loss or slippage can compromise surgical outcomes [3].

A slipped muscle refers to a situation where the muscle retracts posteriorly into its capsule after an attempted reattachment, often due to superficial placement of sutures within the muscle sheath that fail to engage the tendon [3]. Conversely, a lost muscle occurs when both the muscle and its capsule become completely detached from the globe [3]. These complications can result in under correction, overcorrection, diplopia, or restricted eye movement [2]. Immediate and accurate identification and reattachment of the muscle to the sclera is critical for restoring function and alignment [3]. A thorough anatomical understanding of the EOMs is essential for preventing and/or managing such complications. The recti muscles - medial rectus (MR), lateral rectus (LR), superior rectus (SR), and inferior rectus (IR) - originate from the common tendinous ring (annulus of Zinn) and insert anterior to the equator of the globe [4]. The superior oblique (SO) originates from the sphenoid bone and inserts on the superior-posterior nasal quadrant of the globe, after passing through the trochlea [4]. The inferior oblique (IO) arises from the medial orbital wall and inserts on the inferior-posterior temporal quadrant of the globe [4].

These muscles are well-studied, with prior literature providing measurements of their lengths, insertional widths, distances from the limbus, and relationships to adjacent structures [5-15]. While valuable, the applicability of current morphometric data is limited as anatomical variations between ethnic groups have been well documented in the literature [7,8,11-13]. Ethnic variation in orbital anatomy can influence the site and width of muscle insertion as well as muscle length, thereby affecting surgical landmarks and complication risks [7,8,12]. Surgeons must balance insertion displacement with width modification to preserve the desired arc of scleral contact [16]. There is a gap in ethnically diverse anatomical reference data, specifically in Caucasian cohorts. Given this precision requirement, reliable data on Caucasian orbits is essential. Furthermore, though some studies have examined the relationships between oblique and recti muscles, very few dissection studies have specifically analyzed inter-recti distances in the context of aiding identification and repair during strabismus surgery, as well as during required corrective surgery [7,12,13,16]. When a muscle is not clearly visible while correcting these complications, knowing the typical spatial relationship between adjacent recti muscles can help surgeons triangulate and locate the expected position of the missing structure. This anatomical insight can be pivotal in revision surgeries where normal landmarks are obscured.

Aims

This exploratory pilot study aims to recognize the morphometric variation found in the EOMs of Caucasian cadavers and to encourage further studies that can support primary and corrective strabismus surgery.

As a primary target, this study will document a detailed morphometric assessment of the recti and oblique muscles from the assessed Caucasian cadavers and compare obtained recti measurements with data from alternate ethnic populations to highlight the potential for significant inter-ethnic variations.

As a secondary target, this study aims to explore inter-recti distances, an under-researched metric, to establish the potential for a reliable triangulation map for muscle insertion.

This research has been presented in the British Association of Clinical Anatomists (BACA) Winter Scientific Meeting (December 12, 2025) and received positive feedback as an oral presentation.

## Materials and methods

Study design and specimen selection

This cross-sectional anatomical study examined six paired orbits extracted from three formaldehyde-embalmed cadavers of Caucasian descent, aged 86-92 years. All specimens were obtained from the University of Birmingham, Human Anatomy Unit, Department of Biomedical Sciences and pictures were taken using a NIKON^TM^ D7000 camera. Ethical considerations were strictly adhered to and all procedures were conducted in compliance with the Human Tissue Act, 2004. All cadavers examined in this study had provided written consent for their bodies to be used for research after death.

The integrity of the morphometric data was preserved by restricting analysis to cases with anatomically intact orbits and globes, in which the cause of death was unrelated to the ophthalmic or orbital regions. Only specimens without ocular pathology or any documented history of ocular surgery were included.

Cases were excluded if there was a history of eye disease or surgery, if the eyes had been donated, or if there was any evidence of orbital trauma.

Dissection procedure

**Figure 1:**
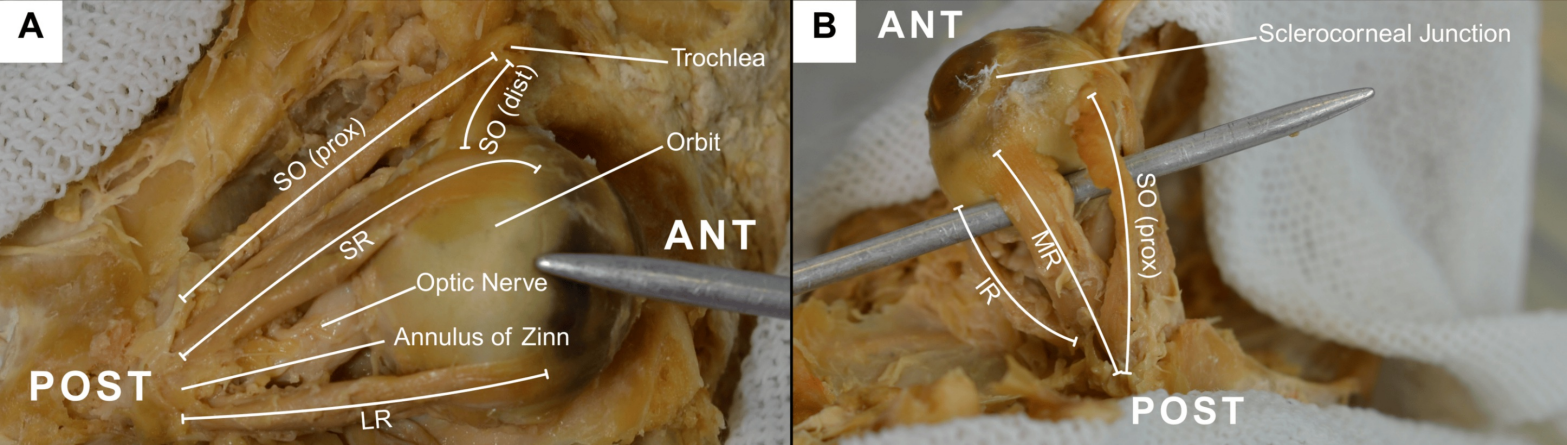
Orbital dissection in situ, demonstrating key anatomical landmarks Superior and medial orbital view prior to complete orbit extraction, noting key anatomical landmarks amongst EOMs. A: SO (prox), SO (dist), SR, LR, Optic Nerve, Annulus of Zinn, Trochlea; Orbit; B: SO, MR, IR, Sclerocorneal Junction SR: Superior rectus; IR: Inferior rectus; LR: Lateral rectus; MR: Medial rectus; SO (prox): Proximal superior oblique; SO (dist): Distal superior oblique; ANT: Anterior orbit; POST: Posterior orbit; EOM: Extraocular muscle; SO: Superior oblique

Superior orbitotomy approach with combined facial and intracranial access was employed to ensure full exposure of the orbital contents. Access was gained by carefully removing orbital roof, followed by the incision and reflection of the periorbita. The levator palpebrae superioris was identified and excised to reveal the deeper EOMs with relevant insertion and origin sites (Figure 1).

Following the exposure of the annulus of Zinn, the EOMs were transected at their origins. The optic nerve was severed proximal to the optic canal to facilitate the safe removal of the globe. The IO was then identified and dissected in situ. Traced from its origin on the anterior medial orbital floor, the IO path was followed beneath the IR to its insertion on the posterolateral globe. Relevant measurements of the IO were noted down.

To mitigate the "collapsed globe" effect common in post-mortem specimens, a post-extraction inflation protocol was implemented. Enucleated globes were inflated until taught with 6-7 ml of ultrasound gel, to restore physiological intraocular volume and mitigate shape distortion caused by fluid egress during handling. All measurements were taken immediately after globe inflation in order to minimize globe shrinkage associated with tissue handling. This critical step ensured that subsequent measurements of scleral curvature and limbal distance remained more representative of in vivo dimensions.

Measurement techniques

All measurements were recorded with a standard metric ruler and rounded to the nearest 1 mm. To accurately capture the three-dimensional geometry of the globe, a non-stretchable string-arc technique was utilized for all curved or irregular distances. The string was meticulously aligned along the contour of the structure (e.g., the scleral arc) and subsequently measured against a linear scale to obtain the "true" anatomical distance. To eliminate inter-rater bias and minimize human error, a triple-observer verification protocol was established. Each parameter was independently measured by all three researchers and the mean value was utilized as the definitive measurement for analysis. Inter-observer reliability was assessed using Lin’s Concordance Correlation Coefficient (ρc). Values >0.8 were considered indicative of good agreement.

Parameters measured

**Figure 2:**
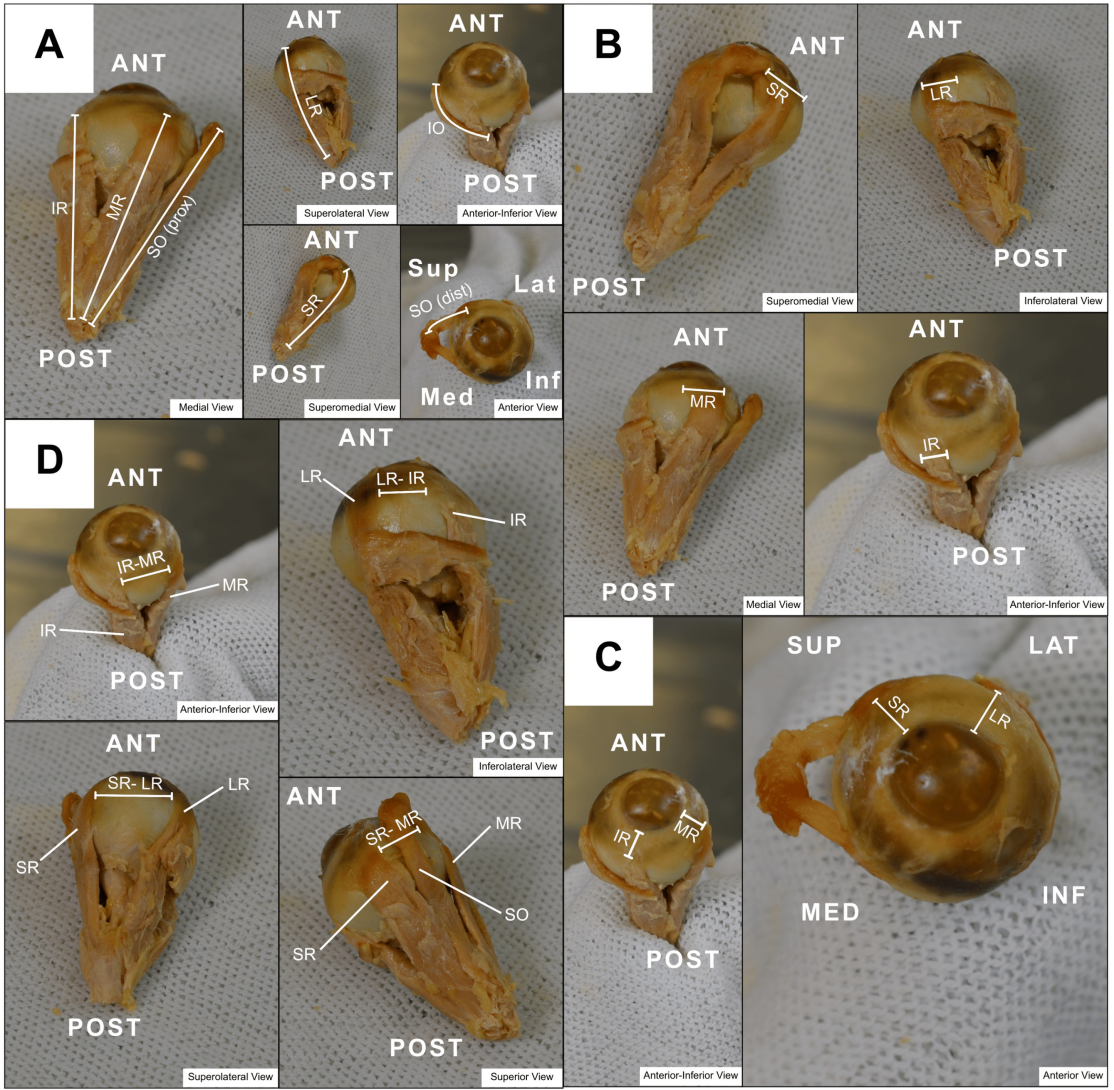
Orbital dissection following globe removal: key anatomical measurements All distances measured on the extracted orbit. Figure 2A: Length of EOMs defined as between the origin and insertion; Medial view: IR, MR, SO (prox); Superolateral view: LR; Anterior-inferior view: Inferior Oblique; Superomedial view: SR; Anterior view: SO (dist). Figure 2B: Width of tendonous insertion for recti muscles. Superomedial view: SR; Inferolateral view: LR; Medial view: MR; Anterior-inferior view: IR. Figure 2C: Distance between end fibers of recti EOMs and sclerocorneal junction; Anterior-inferior view: MR/IR to corneal limbus; Anterior view: SR/LR to corneal limbus. Figure 2D: Distance between outer recti muscle fiber insertions within the tendon sheath; Anterior-inferior view: IR to MR; Inferolateral view: LR to IR; Superolateral view: SR to LR; Superior view: SR to MR. SR: Superior rectus; IR: Inferior rectus; LR: Lateral rectus; MR: Medial rectus; SO (prox): Proximal superior oblique; SO (dist): Distal superior oblique; ANT: Anterior orbit; POST: Posterior orbit; INF: Inferior orbit; SUP: Superior orbit; MED: Medial orbit; LAT: Lateral orbit; EOM: Extraocular muscle

Several morphometric parameters were measured. With the muscle gently pulled taut, the length of each EOM was defined as the maximum distance from its origin to its insertion point, defined as the midpoint between the medial and lateral borders of the tendon at scleral insertion. For the SO, measurements were taken in two segments, proximal and distal to the trochlea (Figure 2A). The width of each rectus muscle insertion was measured as the horizontal distance between the endpoints of the tendinous insertion (Figure 2B). The distance from the limbus to each rectus insertion was recorded as the distance from the midpoint of the muscle insertion to the nearest point of the sclerocorneal junction (Figure 2C). Inter-recti distances were measured as the shortest distance between adjacent rectus tendinous insertions (Figure 2D).

Data analysis

The data was tabulated and analzsed using Microsoft Excel^TM^ 64-bit. Descriptive statistics, including mean values, SDs, and maximum and minimum values, were calculated for all tested variables.

To validate the use of pooled data, the Wilcoxen Signed-Rank Test (P < 0.05) was applied to assess morphological symmetry between paired left and right orbits. Welch’s Unpaired T-Test (P < 0.05) was used to compare the Caucasian morphometric data from this study against previously published data from Thai, Mexican, Indian, and Taiwanese cohorts [7,8,12,13]. This specific t-test was selected to account for unequal variances and sample sizes between the compared studies. Observational comparative analysis was employed for inter-recti distances due to the limited and inconsistent reporting of this parameter in existing literature [7,16].

## Results

The Wilcoxon Signed-Rank Test revealed no statistically significant differences across any of the evaluated anatomical parameters between the bilateral orbits (P > 0.05). This finding of high anatomical symmetry permitted the grouping of data from all six orbits for subsequent descriptive and comparative analysis, thereby strengthening the statistical power of the small cohort.

Descriptive morphometry of Caucasian EOMs

Table 1 provides a comprehensive summary of the descriptive statistics for the measured parameters. Lengths of EOMs increased progressively: IR<IO<MR<LR<SR<SO; with IR being shortest and SO being longest. Analysis of the recti muscles revealed a progressive increase in the distance of insertion from the limbus, following the sequence: LR < MR < IR < SR, thus SR demonstrating the most posterior insertion site. Regarding insertional width, it was smallest for the IR, followed by equal widths for MR and LR, and largest for the SR.

**Table 1:** Summarized anatomical measurements (mm) A summary of the mean, SD, minimum, and maximum values for measurements related to the length and width of the EOMs, the distance between recti insertion and corneal limbus, and the distance between adjacent recti insertions. SR: Superior rectus; IR: Inferior rectus; LR: Lateral rectus; MR: Medial rectus; SO (prox): Proximal superior oblique; SO (dist): Distal superior oblique; EOM: Extraocular muscle

	Length							Width				Limbus				Inter-Recti Distances			
	SR	IR	LR	MR	SO		IO	SR	IR	LR	MR	SR	IR	LR	MR	SR→LR	LR→IR	IR→MR	MR→SR
					Prox	Dist													
Mean	40.83	36.80	40.33	38.67	41.00	22.60	37.80	10.33	6.40	8.50	8.50	9.67	9.33	8.50	8.67	13.33	11.67	11.20	12.25
SD	4.12	3.56	4.27	3.78	3.39	5.94	4.27	2.25	1.14	1.23	1.38	1.86	2.42	2.17	1.75	2.42	1.97	2.49	1.41
Min	34.00	34.00	34.00	33.00	37.00	16.00	33.00	8.00	5.00	7.00	6.00	7.00	7.00	6.00	6.00	9.00	9.00	7.00	11.00
Max	45.00	43.00	45.00	44.00	45.00	31.00	42.00	14.00	8.00	10.00	10.00	12.00	13.00	12.00	11.00	16.00	15.00	13.00	14.00

The inter-recti distances exhibited a consistent trend across the dissected orbits. The IR-MR distance was identified as the smallest surgical corridor, followed by LR-IR and MR-SR, with the SR-LR gap representing the largest distance. Despite these identifiable trends, substantial standard deviations were observed for these parameters, suggesting a higher degree of individual variability in inter-recti spacing compared to limbal distances.

Inter-ethnic comparative analysis

**Figure 3:**
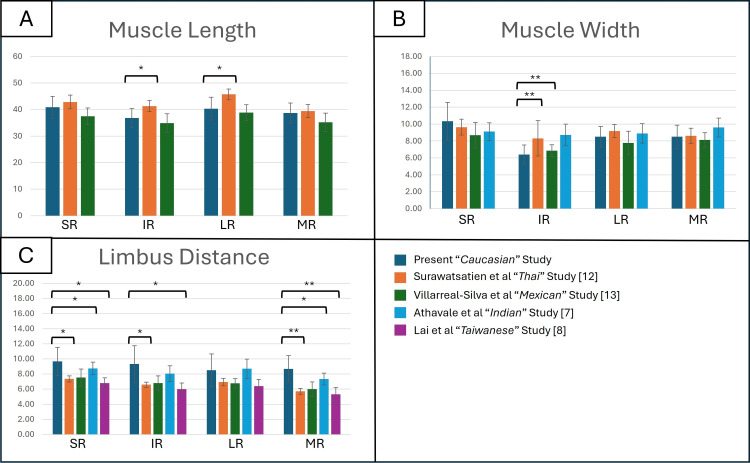
Inter-ethnicity comparison of measured anatomical distances of recti muscles (mm) The bar charts display mean values (±SD) for muscle length (A), muscle width (B), and limbus distance (C) of the SR, IR, LR, and MR muscles across five ethnic groups. SR: Superior rectus; IR: Inferior rectus; LR: Lateral rectus; MR: Medial rectus *: significant difference  (p<0.05); **: very significant difference (p<0.001)

To investigate ethnic variability, morphometric data from the present Caucasian cohort were compared with established data from Thai, Mexican, Indian, and Taiwanese populations using Welch’s Unpaired T-Tests (P < 0.05) (Figure 3) [7,8,12,13].

Significant inter-ethnic differences were identified across multiple parameters. The IR and LR were significantly shorter in the present cohort compared with the Thai population (P < 0.05) [12]. Differences were also observed in insertional width, with the IR demonstrating a markedly narrower insertion relative to both Thai and Mexican cohorts, reaching a high level of statistical significance (P < 0.001) [12,13].

Limbal distances in the present study were consistently greater than those reported in East Asian populations. The SR insertion was positioned more posteriorly, with a mean distance of 9.67 mm (SD ±1.86), showing significant differences compared with Thai, Indian, and Taiwanese cohorts (P < 0.05). The IR similarly demonstrated a more posterior insertion at 9.33 mm (SD ±2.42), significantly greater than values reported in Thai and Taiwanese studies (P < 0.05). The MR showed a mean limbal distance of 8.67 mm (SD ±1.75), with significant posterior displacement relative to the Indian cohort (P < 0.05) and highly significant displacement when compared with Thai and Taiwanese populations (P < 0.001).

Comparison of inter-recti metrics

Inter-recti insertion distances from the present study were compared with the limited body of literature available to the authors, including studies by Athavale et al. and Apt et al., and the historical, cross-referenced findings of Duke-Elder et al. and Renard et al. (Table 2) [7,16]. Due to the absence of granular statistical reporting in the Duke-Elder and Renard series, a formal meta-analytical comparison was precluded.

**Table 2:** Mean inter-recti distances from different morphological studies (mm) Mean inter-recti distances between EOMs from the present study compared with previous morphological studies. Distances are given as mean ± SD where available. Data attributed to Duke-Elder et al. and Renard et al. are historical values reported secondarily in Apt et al. (i.e., not obtained from the original primary publications). “No findings” indicates that no corresponding measurement was reported. SR: Superior rectus; IR: Inferior rectus; LR: Lateral rectus; MR: Medial rectus; EOM: Extraocular muscle

	Present Study	Athavale et al. [7]	Apt et al. [16]	Duke-Elder et al. (as cited in Apt et al. [16])	Renard et al. (as cited in Apt et al. [16])
MR–IR	11.20 +/- 2.49	7.60 +/- 1.88	5.90 +/- 0.80	5.50	(No findings)
IR–LR	11.67 +/- 1.97	8.00 +/- 1.36	8.00 +/- 0.80	7.00	9.00
LR–SR	13.33 +/- 2.42	8.07 +/- 1.36	7.10 +/- 0.80	6.50	8.00
SR–MR	12.25 +/- 1.41	8.85 +/- 1.04	7.50 +/- 0.80	7.00	8.00

Observational comparison revealed substantial global variation across these cohorts [7,16]. While the current Caucasian specimens exhibited larger inter-recti distances than previously reported, no consistent anatomical pattern emerged across the disparate groups, other than MR-IR being consistently the smallest distance. The overall lack of uniformity emphasizes the necessity for further population-specific research to validate these findings and to establish reliable morphometric standards for Caucasian orbits.

## Discussion

Studying the morphometry of human EOMs offers key insights into eye movement and alignment, aiding corrective ocular surgery after a slipped or lost muscle. The present study found no statistically significant differences between the left and right orbits, supporting pooled analysis by confirming bilateral anatomical symmetry at the measured level.

Muscle length

Anatomical evaluation of the Caucasian orbits revealed that SO was the longest EOM, followed by the SR, LR, MR, IO, and IR. Consistent with the Thai and Mexican studies, LR and SR were the longest recti muscles, while MR and IR were the shortest [12,13]. This strongly suggests that, in Thai, Mexican and Caucasian cohorts, the superolateral portion of the cone surrounding the globe is large, making it a safer entry route for lost muscle retrieval during corrective surgery [13]. In the context of "lost" muscle retrieval, knowledge of the relative anatomical lengths of EOMs is helpful for locating a retracted muscle belly [12].

Insertional muscle width

The SR exhibited the widest insertion, followed by the LR, MR, and IR. This sequence aligns with Thai and Mexican studies but slightly deviates from Indian cohorts, which report the MR as having the widest insertion [7,12,13]. Most notably, we observed a highly significant decrease in IR width in Caucasians compared to Thai and Mexican data [12,13].

For the prevention of slipped muscles, this width data is clinically transformative. A narrower IR tendon in Caucasians reduces the margin of error for suture placement; failure to adjust for this narrower target can result in "superficial capture," where the suture engages only the thin muscle sheath rather than the robust tendon. Because recti width directly influences contractile force and focality, optimizing the reattachment width is also essential for restoring physiological recti strength and preventing post-operative under-correction [17].

Differences in tendon width between antagonistic vertical (SR vs IR) and horizontal (LR vs MR) muscles can affect postoperative vertical, horizontal, or torsional balance. The average width difference between antagonistic muscles, specifically the 3.93 mm gap between the mean SR and IR in the present study, is markedly higher than the 0.39 mm (Indian study), 1.83 mm (Mexican study), and 1.33 mm (Thai study) differences reported in the compared ethnicities [7,8,12,13]. No difference was observed in mean LR-MR tendon widths in this study, as compared to 0.37 mm (Mexican study), 0.56 mm (Thai study), and 0.70 mm (Indian study) in the compared ethnicity studies [7,8,12,13]. This notable difference in observed antagonistic muscle widths in Caucasians relative to other ethnicities further emphasizes the need of population-specific optimization of insertion width to restore vertical and torsional equilibrium.

Limbal distance

The possibility of posterior-shifted limbal distances in Caucasian patients was noted in three of the four recti muscles when compared to the East Asian (Thai and Taiwanese) populations (Figure 3) [8,12].

It is also important to note that the Taiwanese measurements were taken "in vivo," whereas the present study examined formalin-embalmed cadavers, in which tissue shrinkage is expected [8,18,19]. Despite this, the Caucasian orbits in the current study reported larger limbal distances for the SR, MR, and IR compared with the Taiwanese cohort.

In Lai et al.’s cohort comparison against the Apt et al. cadaveric study, limbal distances were significantly larger in three of the four recti muscles (SR, LR and IR) in the Apt et al. cohort, which consisted predominantly (however not entirely) of Western cadavers [8,16]. Consistent with Apt et al. findings, the Taiwanese study also reported no correlation between axial length and EOM insertion sites [8,16]. This further supports the possibility reflected in the present study, that ethnic variation may contribute to the larger limbal distances observed in Caucasian compared to East Asian orbits.

As in delayed revision surgeries, where the original insertion may be obscured by fibrosis and scar tissue, an accurate knowledge of relative rectus insertions would prove useful in restoring binocular vision [20]. The present study demonstrated an ascending limbal distance order of LR, MR, IR, SR, whereas the historically accepted Spiral of Tillaux describes MR, IR, LR, SR - an order mirrored by the Indian, Thai, and Taiwanese studies [7,12,21]. Interestingly, the Mexican cohort also deviated from the historical standard, exhibiting a sequence of MR, LR, IR, SR [13]. The observation that rectus muscle insertions do not consistently follow the Spiral of Tillaux indicates that over-reliance on traditional, spiral-based “triangulation” rules could lead to surgical disorientation. Hence, other metrics such as the proposed inter-recti distances, should be considered for accurate reattachment.

Inter-recti spacing

The present study, like Apt et al., also assessed inter-recti distances, a heavily underexplored variable [16]. It demonstrated the ascending inter-recti order of: IR-MR as the smallest, followed by LR-IR, then MR-SR and finally SR-LR as the largest distance. Observational literature comparison confirmed IR-MR as the relative shortest distance, though ranking beyond this varies between studies [7,16]. Inter-recti comparisons with Athavale et al., Apt et al., Duke-Elder et al., and Renard et al. revealed broad variability and lacked a consistent anatomical pattern, complicating the formulation of clinical conclusions [7,16].

With further research, this spatial data can provide a "secondary map" for surgeons: if a muscle is retracted and the scleral tissue is scarred, measuring the inter-recti distance from a stable adjacent muscle can help the surgeon to strategically locate the missing insertion site.

Limitations

This study is inherently limited by its small pilot sample size (N=6 orbits), which restricts statistical power and limits the generalizability of the findings. While the use of Welch’s Unpaired T-Test and Wilcoxon Signed-Rank Test helped mitigate the effects of a small cohort by accounting for variance, these results should be interpreted as a pilot morphometric profile. The advanced age of the cadaveric donors (86-92 years) also warrants caution, as it may not perfectly represent the pediatric or adolescent populations more common in primary strabismus cases.

Additionally, data regarding refractive error, axial length, and overall globe dimensions were not available for the cadaveric specimens. These factors may influence EOM positioning and insertional relationships and, therefore, represent potential confounding variables.

Furthermore, methodological differences across studies, such as our use of a non-stretchable string to account for scleral curvature versus the linear calipers used in other studies, may account for some of the reported variation. Additionally, while we utilized ultrasound gel inflation to counteract formalin-related shrinkage and restore physiological volume, not all comparative studies employed such adjustments, potentially skewing inter-study validity.

## Conclusions

This cadaveric pilot study provides an important starting point for optimizing future research on EOM morphometry in Caucasian populations. It does so by comparing specific features of Caucasian orbits with established morphometric data from alternate ethnicities and by promoting the quantification of inter-recti spacing, which can be a valuable tool for immediate surgical utility. The significant deviations from the Spiral of Tillaux together with the marked inter-ethnic variations in muscle length, width and limbal distance, underscore a critical clinical reality: ophthalmic surgery cannot rely on a single anatomical archetype. Further integration of enhanced Caucasian-specific metrics may improve surgeon’s ability to locate, retrieve, and reattach slipped or lost muscles with greater accuracy. Given the exploratory nature and small sample size of this pilot cohort, these findings should be interpreted as preliminary and require validation in larger, multi-center studies before definitive clinical application. This study underscores the need for standardized, demographic-specific normative data to enhance surgical planning and optimize long-term patient outcomes in primary and corrective strabismus surgery.
